# Reassessing Revascularization Strategies in Coronary Artery Disease and Type 2 Diabetes Mellitus

**DOI:** 10.3389/fcvm.2021.738620

**Published:** 2021-10-21

**Authors:** Bo Liang, Xin He, Ning Gu

**Affiliations:** ^1^Nanjing University of Chinese Medicine, Nanjing, China; ^2^Nanjing Hospital of Chinese Medicine Affiliated to Nanjing University of Chinese Medicine, Nanjing, China

**Keywords:** coronary artery disease, type 2 diabetes mellitus, coronary artery bypass surgery, percutaneous coronary intervention, adverse clinical outcomes

## Abstract

Percutaneous coronary intervention (PCI) or coronary artery bypass grafting (CABG) is still controversial in patients with coronary artery disease (CAD) and type 2 diabetes mellitus (T2DM). Here, we aimed to evaluate the long-term follow-up events of PCI and CABG in these populations. Relevant randomized controlled trials were retrieved from PubMed, Embase, and the Cochrane databases. The pooled results were represented as risk ratios (RRs) with 95% confidence intervals (CIs) with STATA software. A total of six trials with 1,766 patients who received CABG and 2,262 patients who received PCI were included in our study. Patients in the CABG group were significantly associated with a lower all-cause mortality compared with those in the PCI group (RR = 0.74, 95% CI = 0.56–0.98, *P* = 0.037). Cardiac mortality, recurrent myocardial infarction, and repeat revascularization were also significantly lower in the CABG group (RR = 0.79, 95% CI = 0.40–1.53, *P* = 0.479; RR = 0.70, 95% CI = 0.32–1.56, *P* = 0.387; and RR = 0.36, 95% CI = 0.28–0.46, *P* < 0.0001; respectively). However, compared with the PCI group, the cerebral vascular accident was higher in the CABG group (RR = 2.18, 95% CI = 1.43–3.33, *P* < 0.0001). There was no publication bias in our study. CABG revascularization was associated with significantly lower long-term adverse clinical outcomes, except cerebral vascular accident, compared with PCI in patients with CAD and T2DM.

**Systematic Review Registration:** PROSPERO, identifier: CRD42020216014.

## Introduction

Cardiovascular disease is the leading cause of death in the world (1). According to World Health Organization, an estimated 17.3 million people died from cardiovascular diseases in 2008, accounting for 30% of the global deaths. It is predicted that by 2030, about 23.6 million people will die from cardiovascular diseases, mainly coronary artery disease (CAD) and stroke. Risk factors for CAD include smoking, unhealthy diet, inadequate daily exercise, overweight, or obesity (1, 2), which are also risk factors for diabetes (3). Diabetes poses as a major risk factor for the development of cardiovascular disease, which ultimately results in being the most common cause of death in those with diabetes (4). Diabetes is caused by insulin produced by the pancreas or tissue resistance in the terminal organs, manifested as hyperglycemia or elevated glycosylated hemoglobin A1C (3). Type 2 diabetes mellitus (T2DM) is the most common form of diabetes, accounting for 90–95% of the diagnosis of diabetes, and continues to grow rapidly around the world (5). Due to the few symptoms or signs of early T2DM, about half of the diabetics do not know that they have the disease. Symptoms are ignored before diagnosis and thus lead to diabetic complications, which can lead to cardiovascular diseases (6).

There is a strong correlation between CAD and T2DM (7, 8). Compared with the non-diabetic population, the progress of atherosclerosis in the diabetic group is earlier and more severe (9–11). Additionally, more complex coronary anatomy usually emerges in the diabetic group, which challenges the revascularization (12), whether coronary artery bypass grafting (CABG) or percutaneous coronary intervention (PCI). Cardiovascular deaths account for 52% of deaths in T2DM (13, 14). Moreover, T2DM increases the risk of cardiovascular death by two to six times (3). The mortality of diabetic patients after myocardial infarction is also significantly higher than that of non-diabetic patients (15, 16). Compared with non-diabetic patients of the same age group, the cardiovascular mortality of patients with no other traditional cardiovascular risk factors increased by 4.4 times (17, 18). Thereby, T2DM imperceptibly increases CAD mortality (19, 20). Although the mortality of CAD has been well-controlled with the development of interventional strategies (21), the prognosis of patients with CAD and T2DM is still very poor (22). One of the reasons is that diabetic patients have a worse prognosis following revascularization treatment (23). Simultaneously, patients with T2DM are at an increased risk of having a cardiovascular event, and more likely to have diffuse and multivessel vascular lesions (24, 25). Such patients are prone to a more rapid progression of atherosclerosis, significantly increasing the need for myocardial revascularization (26). Besides, patients with T2DM also have a worse prognosis following a coronary revascularization procedure (23). In this population, it may be difficult to choose the optimal revascularization strategy. The outcomes of different revascularization strategies have been extensively evaluated (27), but comparative data on the cause of mortality after these revascularization procedures are limited. A previous study suggests that, for patients with insulin-treated T2DM and multivessel ischemic heart disease, CABG is usually superior to PCI, leading to lower rates of all-cause mortality, major adverse cardiovascular, cerebrovascular events, and repeat revascularization in the long term, but the higher rate of stroke in the CABG group (28). It is necessary for further researches with a larger number of randomized patients to completely solve this issue. Therefore, we conducted this metaanalysis of randomized controlled trials (RCTs) to assess whether CABG can reduce adverse clinical outcomes in this special population and to determine the more suitable revascularization strategy.

## Materials and Methods

This study adhered to the PRISMA guidelines (29) and registered at PROSPERO with a unique identifier CRD42020216014.

### Data Sources

Two reviewers (BL and XH) searched several electronic databases, including PubMed, Embase, and the Cochrane databases, along with RCTs from inception until July 2020, using the Medical Subject Heading and the keyword search terms: “coronary artery disease,” “diabetes mellitus type 2,” “percutaneous coronary intervention,” and “coronary artery bypass grafting.” To further enhance this search, the relevant abbreviations, such as CAD, T2DM, CABG, and PCI, were also conducted. References were also checked for potential RCTs and there was no language restriction.

### Selection Criteria

We only included RCTs comparing long-term (more than 1 year) adverse clinical outcomes of different revascularization therapies, either CABG or PCI, in patients with CAD and T2DM. When the study was published repeatedly, the latest or complete data were included (30).

### Interventions

Patients with T2DM who received insulin or medication were included in the study. These patients randomly underwent revascularization by either CABG or PCI. We evaluated the quality of the included studies based on the adequate description of treatment allocation and blinded outcome assessment.

### Outcomes and Definitions

All-cause mortality during a long-term follow-up period was considered the primary outcome. Secondary outcomes for this study were composite cardiac mortality, recurrent myocardial infarction (MI), cerebralvascular accident (CVA), and repeat revascularization.

### Data Extraction and Quality Assessment

Two reviewers (XH and BL) independently assessed study eligibility and extracted data. We used a standardized data collection form to objectively evaluate each included study (30). The third reviewer (NG) solved the disagreement (31, 32). The extracted data included the year of publication, sample size, duration of follow-up, and the clinical outcomes (including all-cause mortality, recurrent myocardial infarction, CVA, and repeat revascularization). The bias risk was assessed using the components recommended by the Cochrane Collaboration guidelines, as described previously (31).

### Statistical Analysis

This study was performed using STATA software (version 15, USA). Risk ratios (RRs) and 95% confidence intervals (CIs) were used as summary statistics. Statistical heterogeneity was assessed for each outcome using the *I*^2^ statistic. *I*^2^ <25% is low heterogeneity, higher than 75% is high heterogeneity, and between the two is moderate heterogeneity, as described previously (31, 33–35). If *I*^2^ was <50%, the fixed-effect model of Mantel–Haenszel was used to assess the overall estimate, otherwise, a random-effect model was conducted to calculate the pooled RRs (34). Moreover, sensitivity analysis (30), L'Abbe plot (36, 37), and Galbraith radial plot (38) were conducted to assess heterogeneity. Lastly, the funnel plot and Begg's and Egger's tests were implemented to assess the publication bias.

## Results

### Characteristics of Included Studies

**Figure 1:**
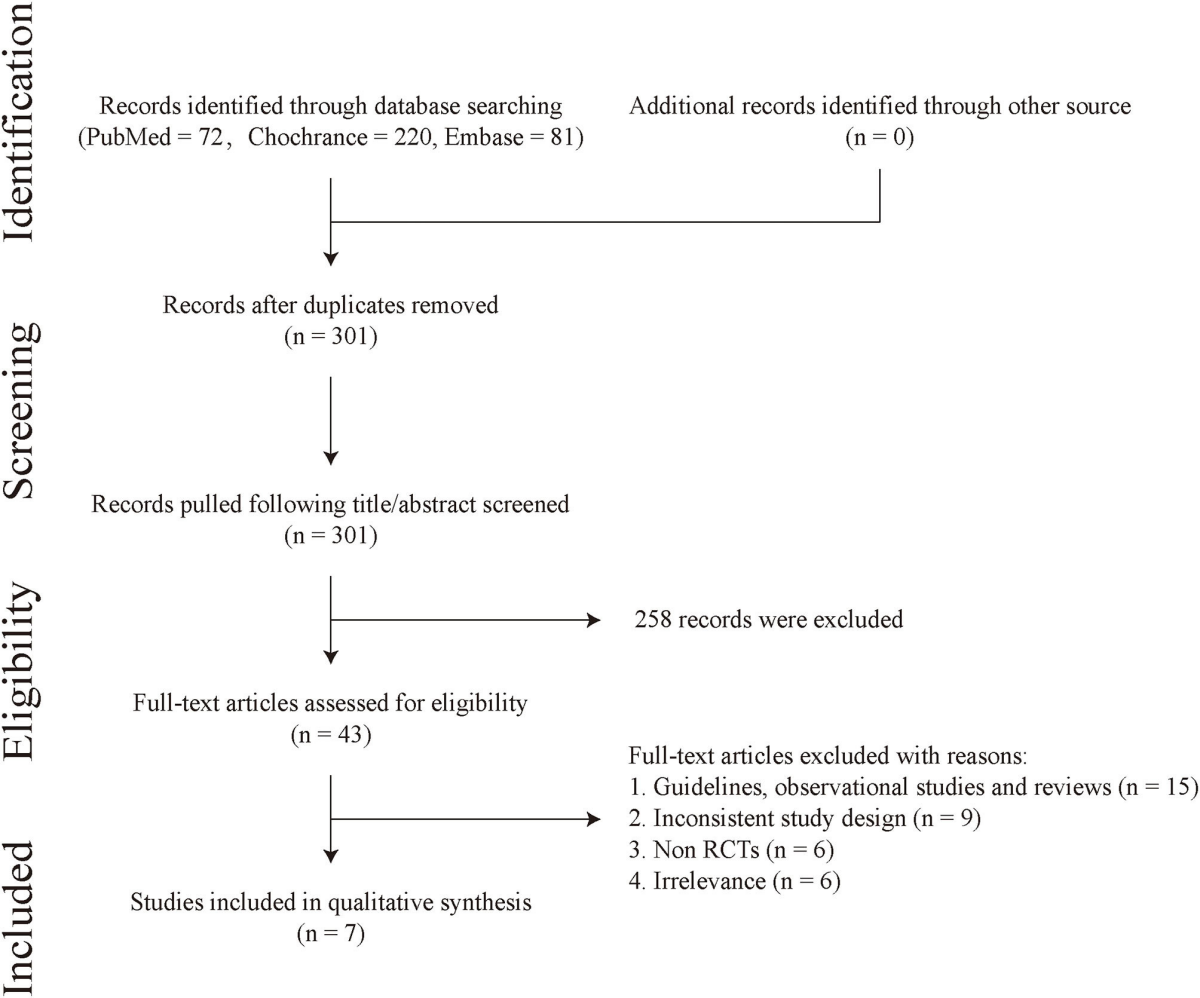
Flow diagram.

Our search identified 373 articles, ultimately six RCTs [ARTS (39), BARI 2D (40), FREEDOM (41)/FREEDOM Follow-On (42), MASS II (43), SYNTAX (44), and VACARDS (45)] were included in this study. The flow diagram of this study selection is represented in Figure 1. A total of 4,028 patients underwent revascularization, among them 2,262 patients were assigned to the PCI group and 1,766 patients were assigned to the CABG group. Most trials were international RCTs. The characteristics of the included studies are presented in Table 1, and the baseline clinical characteristics are shown in Supplementary Table 1. Since FREEDOM Follow-On (42) was the longer follow-up data of FREEDOM (41) and only reported all-cause mortality, we used data from FREEDOM Follow-On (42) to analyze all-cause mortality.

**Table 1:** Baseline patient characteristics.

**Trials**	**Year**	**Numbers (CABG/PCI) (n)**	**Ages (years)**	**Males (%)**	**Outcomes**	**Follow-up (year)**	**References**
ARTS	2001	96/112	62.6/62.4	148 (71.12%)	a, b, c, d	1	(39)
BARI 2D	2009	378/798	Not applicable	Not applicable	a, e	5	(40)
FREEDOM	2014	277/325	61.9 ± 9.2/63.2 ± 9.2	369 (61.30%)	a, b, d, f	1,5	(41)
FREEDOM Follow-On	2019	947/953	63.3	1356 (71.37%)	a	8	(42)
MASS II	2013	80/64	59 ± 8/61 ± 9	128 (88.89%)	a, e	10	(43)
SYNTAX	2013	221/231	65.4 ± 9.2	321 (71.02%)	a, b, d, e, f	5	(44)
VACARDS	2013	97/101	62.1 ± 7.4/62.7 ± 7.1	196 (98.99%)	a, b	1,2	(45)

*a, all-cause mortality; b, myocardial infarction; c, cerebralvascular accident; d, repeat revascularazition; e, cardiac mortality; f, stroke*.

### Primary Outcome

**Figure 2:**
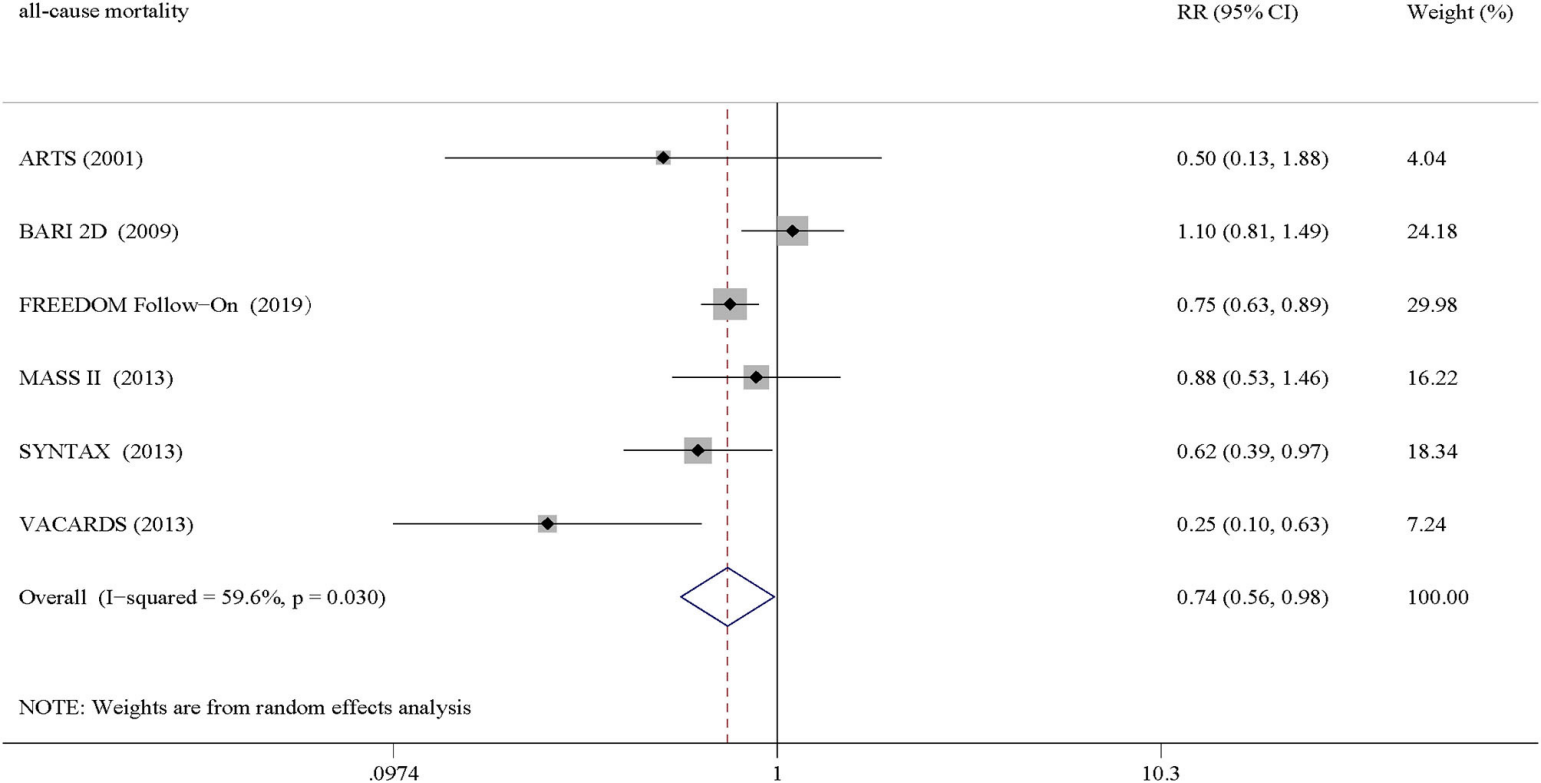
Pooled results of all-cause mortality.

All included studies reported all-cause mortality. The all-cause mortality of CABG was significantly lower than that of PCI in patients with CAD and T2DM (RR = 0.74, 95% CI = 0.56–0.98, *P* = 0.037), albeit with moderate heterogeneity (*I*^2^ = 59.6%, *P*_h_ = 0.030) (Figure 2).

### Heterogeneity and Publication Bias

**Figure 3:**
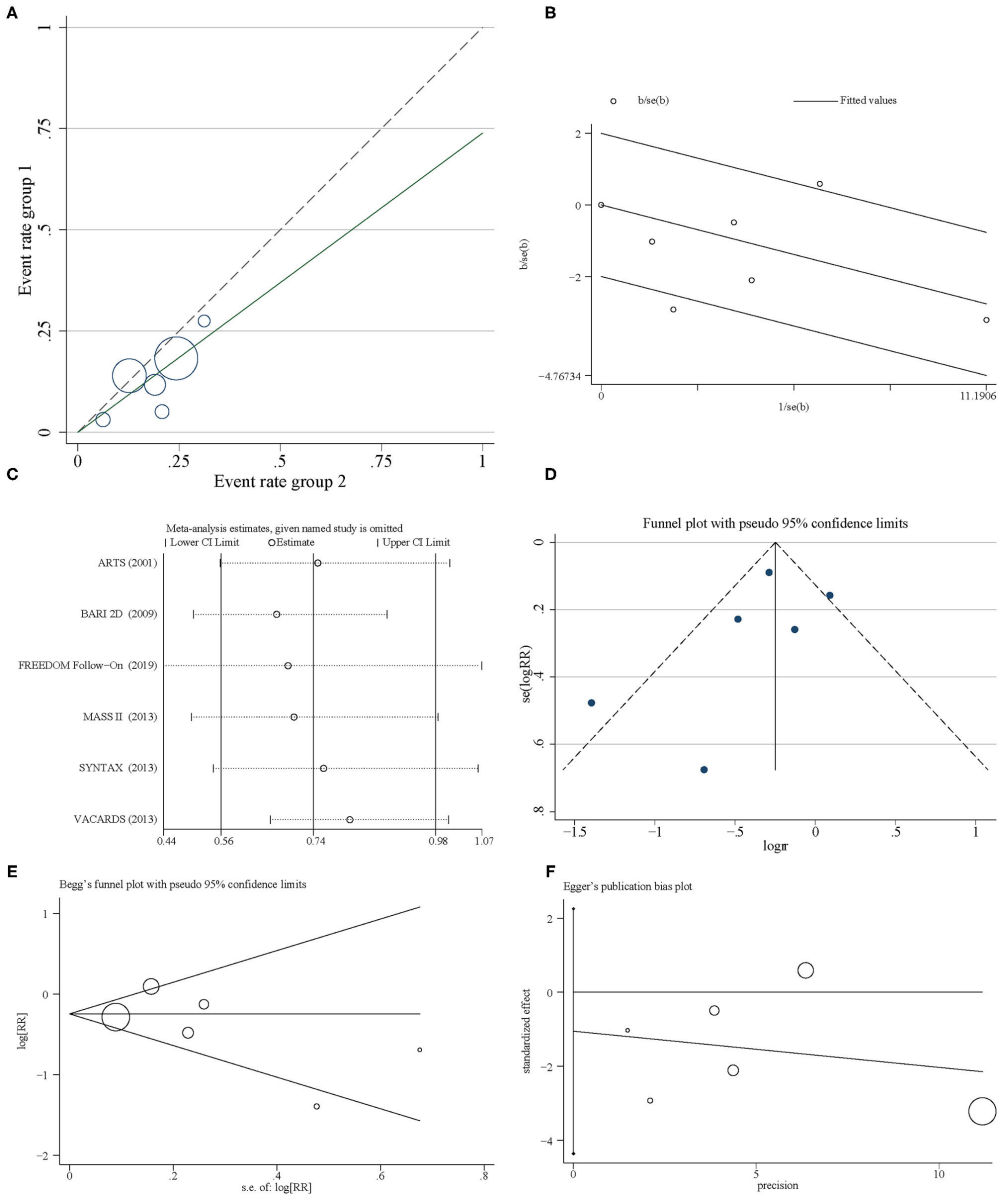
Heterogeneity and sensitivity analysis. **(A)** L'Abbe plot. **(B)** Galbraith Radial plot. **(C)** Sensitivity analysis. **(D)** Funnel plot. **(E)** Begg's plot. **(F)** Egger's plot.

Further performances of the L'Abbe plot (Figure 3A) and Galbraith Radial plot (Figure 3B) indicated that there was a possible heterogeneity in this pooled result. Therefore, a search for heterogeneous sources was needed. Sensitivity analysis was performed to evaluate individual study's influence on the pooled results to verify the consistency of the meta-analysis consequences. The results revealed that FREEDOM Follow-On (42) might have a greater impact on heterogeneity, which disclosed that they may be the source of heterogeneity (Figure 3C). However, when FREEDOM Follow-On was omitted, the pooled results did not change (RR = 0.69, 95% CI = 0.44–1.07) (Figure 3C). Funnel plot analysis showed that there was no statistical evidence of publication bias of all-cause mortality in this study (Figure 3D). Moreover, Begg's and Egger's tests were applied to confirm this (*P*_Begg's test_ = 0.707 and *P*_Egger's test_ = 0.427, respectively) (Figures 3E,F).

### Secondary Outcomes

**Figure 4:**
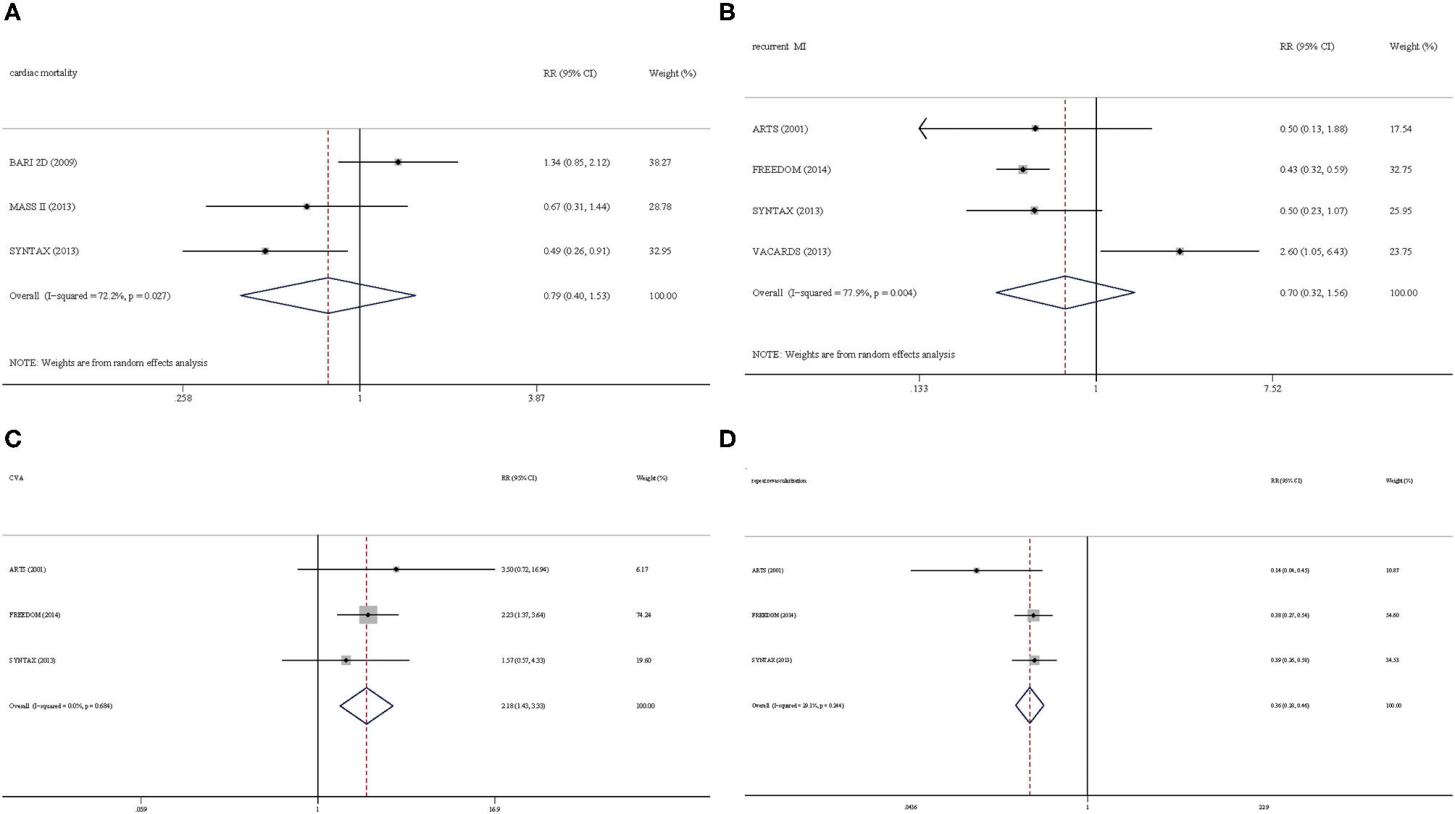
Secondary outcomes. **(A)** Cardiac mortality. **(B)** Recurrent myocardial infarction. **(C)** Cerebralvascularaccident. **(D)** Repeat revascularization.

A total of three trials reported cardiac mortality. We found that cardiac mortality of CABG was lower than that of PCI in patients with CAD and T2DM, with no statistical difference (RR = 0.79, 95% CI = 0.40–1.53, *P* = 0.479) (Figure 4A). Data synthesis of four trials showed that recurrent MI was more favorable in the CABG group than PCI group (RR = 0.70, 95% CI = 0.32–1.56, *P* = 0.387) (Figure 4B). Moreover, patients in the PCI group had more CVA than those in the CABG group (RR = 2.18, 95% CI = 1.43–3.33, *P* < 0.0001) (Figure 4C), whereas, patients in the CABG group had a lower repeat revascularization than those in the PCI group (RR = 0.36, 95% CI = 0.28–0.46, *P* < 0.0001) (Figure 4D).

## Discussion

Previous studies have compared the effects of CABG with PCI on health-related quality of life in patients with CAD with multivessel disease. In general, CABG provides better relief of central colic in the first 1–3 years after initial revascularization than PCI (46). However, with the advancement of revascularization technology, the benefits provided by CABG compared with PCI are gradually reduced, but it has a higher rate of stroke. Recent studies comparing CABG with PCI found significantly lower mortality rates among patients with T2DM revascularized by CABG compared with those patients revascularized by PCI, but a significantly higher risk of stroke in these studies, with no statistical significance (28, 47, 48). Besides, Gargiolo et al. compared the 5 years clinical outcomes and showed that the rate of repeated revascularization was significantly increased in the PCI group, but there was no statistical difference in mortality, MI, and stroke between CABG and PCI (49). These studies showed that data regarding the long-term adverse clinical outcomes in patients with T2DM revascularized by either CABG or PCI are still controversial (50). So, we aim to solve this issue in our present study.

In this study, we compared the effects of two different revascularization strategies, CABG and PCI, on CAD patients with T2DM. Our results showed that an all-cause mortality, cardiac mortality, recurrent MI, and repeat revascularization were lower in the CABG group when compared with the PCI group, whereas CVA was higher in the CABG group compared with the PCI group, with a statistical significance in the present study. Therefore, CABG is the first choice for most patients with CAD patients with T2DM. However, longer-term follow-up and data from more trials will be needed to provide a more precise comparison of the efficacy of these two revascularization strategies for this particular population. CARDia is the first RCT of coronary revascularization in diabetic patients, but the 1-year results did not show that PCI is non-inferior to CABG (51). In the total 510 patients, 4.90% were type 1 diabetes mellitus (17 and eight cases in the CABG group and PCI group, respectively). Although the number of cases is small, we can only approximately infer that the results of this trial can be applied to T2DM patients, and we have not included the analysis of CARDia in our study. It is not a unique instance, but has its counterpart. We also cannot extract the data of CAD patients with T2DM in SOS (the Stent or Surgery trial) (52), ERACI II (Argentine randomized study: Coronary angioplasty with stenting vs. coronary bypass surgery in patients with multiple-vessel disease) (53), and ASAN-MAIN (ASAN medical center-left MAIN revascularization) (54).

For the treatment of unprotected left main CAD, PCI with stent implantation showed similar long-term mortality and rates of death, Q-wave MI, or stroke. However, stenting, even with drug-eluting stents, was associated with higher rates of repeat revascularization than was CABG. In the Intermountain Heart Registry of patients undergoing revascularization for multivessel CAD, a long-term benefit was found, in relation to both death and major adverse cardiovascular events, for CABG over PCI regardless of diabetic status (55). However, in ARTS-II (arterial revascularization therapies study-part II) at 3-year follow-up, PCI using sirolimus-eluting stents for patients with multivessel CAD appears to be a valuable alternative to CABG for both diabetic and non-diabetic patients (56), and in the 5-year follow-up, PCI using sirolimus-eluting stents had an approximately safer record and higher MACCE rate compared with CABG (57). In addition to multivessel CAD, recent observational and subgroup analyses suggest that CABG might be the preferential method of revascularization for patients with T2DM and MVD, also in the non-ST-segment elevation acute coronary syndrome setting (58). There are many uncertainties regarding the best revascularization strategy in the multivessel CAD or acute scenario, and dedicated randomized clinical trials are needed.

There existed several limitations in our work that need to be optimized in the future. First, to assess the long-term follow-up events between CABG and PCI in patients with CAD and T2DM, we only included six RCTs after strict inclusion and exclusion criteria, indicating we may be missing some important evidence from observational studies. Moreover, differences in procedural aspects, post-procedural management, and follow-up protocol may have existed between the included trials. In addition, our primary outcome, the all-cause mortality, which is the most comprehensive and unbiased endpoint for myocardial revascularization trials (59, 60), was reported by all the trials. However, our secondary outcomes were not reported by several trials. Fourth, since we cannot obtain the drug use of the included patients, we cannot analyze whether the drug use, especially the hypoglycemic drugs that gradually show cardiovascular benefits (61–64), brings additional benefits in different revascularization strategies. Fifth, the follow-up in each study was different (Table 1), FREEDOM Follow-On and MASS II were followed up for more than 7 years, whereas ARTS and VACARDS were followed up for <3 years. Longer follow-up may show more outcome events, which need to be verified in more carefully designed trials. Finally, although most of the included RCTs were international studies, background heterogeneity cannot be avoided.

## Conclusions

Patients with CAD and T2DM undergoing CABG surgery have lower all-cause mortality, cardiac mortality, recurrent MI, and repeat revascularization, but higher CVA than those undergoing PCI. This information may be useful in counseling patients with T2DM requiring appropriate coronary revascularization; however, more evaluations in adequately powered large trials are required to further confirm the clinical benefit of this strategy.

## Data Availability Statement

The original contributions presented in the study are included in the article/Supplementary Material, further inquiries can be directed to the corresponding author.

## Author Contributions

BL and NG conceived, designed, or planned the idea. BL drafted the manuscript. NG revised the manuscript. All authors collected, analyzed, interpreted data, and read and approved the final manuscript.

## Funding

This study was partly funded by Research and Practice Innovation Plan for Postgraduates of Jiangsu, China (KYCX21_1641), National Natural Science Foundation of China (81774229), Jiangsu Leading Talent Project of Traditional Chinese Medicine (Jiangsu TCM 2018 No. 4), and Jiangsu Universities Nursing Advantage Discipline Project (2019YSHL095).

## Conflict of Interest

The authors declare that the research was conducted in the absence of any commercial or financial relationships that could be construed as a potential conflict of interest.

## Publisher's Note

All claims expressed in this article are solely those of the authors and do not necessarily represent those of their affiliated organizations, or those of the publisher, the editors and the reviewers. Any product that may be evaluated in this article, or claim that may be made by its manufacturer, is not guaranteed or endorsed by the publisher.

## Abbreviations

CABG: coronary artery bypass grafting
CAD: coronary artery disease
CI: confidence interval
CVA: cerebralvascular accident
MI: myocardial infarction
PCI: percutaneous coronary intervention
RCT: Randomized Controlled Trial
RR: Risk ratio
T2DM: type 2 diabetes mellitus.
