# Supplementary material for: Reassessing Revascularization Strategies in Coronary Artery Disease and Type 2 Diabetes Mellitus
Source: Front Cardiovasc Med. 2021 Oct 21;8:738620. doi: 10.3389/fcvm.2021.738620 (PMC8568070; doi:10.3389/fcvm.2021.738620)
Supplement: Supplementary file 1 [file Table_1.pdf]

Supplementary Table 1. Baseline clinical characteristics

Characteristics	ARTS		FREEDOM		FREEDOM Follow-On		MASS II		VACARDS	
	CABG (N=96)	PCI(N=112)	CABG (N=277)	PCI (N=325)	CABG (N=947)	PCI (N=953)	CABG (N=59)	PCI (N=56)	CABG (N=97)	PCI (N=101)
Previous myocardial infarction	47(49%)	46(41%)	71(26%)	83(26%)	237(25%)	250(26%)	22(37%)	26(46%)	34(35%)	45(45%)
Previous stroke			5(2%)	18(6%)	28(3%)	37(4%)			8(8%)	7(7%)
Hypertension	54(56%)	72(64%)	245(88%)	282(87%)	806(85%)	805(84%)	43(73%)	41(73%)	90(93%)	97(96%)
Hypercholesterolemia	47(49%)	62(55%)								
Congestive heart failure			101(36%)	92(28%)					44(45%)	37(37%)
Acute coronary syndrome			99(36%)	112(34%)	279(29%)	304(32%)				
Current smoking	16(17%)	23(21%)	49(18%)	59(18%)	157(17%)	141(15%)			20(21%)	28(28%)
Left ventricular ejection fraction, %	60.3	60.5	66.1±11.1	65.3±12.5	66.7±10.5	65.7±12.1	69±8	68±11		

Values are N (%) or mean ± standard deviation.
